# Work related stress and associated factors among Huajian shoe manufacturing employees in Dukem town, central Ethiopia

**DOI:** 10.1186/s13104-018-3727-5

**Published:** 2018-08-24

**Authors:** Morke Mezgebu Etefa, Mulat Gebrehiwot Teklu, Destaw Fetene Teshome

**Affiliations:** 1 Dukem Town Administration Health Office, Oromia National Regional State, Dukem, Ethiopia; 2 Department of Environmental and Occupational Sciences, Institute of Public Health, College of Medicine and Health Sciences, University of Gondar, Gondar, Ethiopia; 3 Department of Epidemiology and Biostatistics, Institute of Public Health, College of Medicine and Health Sciences, University of Gondar, Gondar, Ethiopia

**Keywords:** Work related stress, Shoe manufacturing employees, Ethiopia

## Abstract

**Objective:**

The objective of this study was to assess the prevalence of work-related stress (WRS) and its determinants among Huajian shoe manufacturing company employees in Dukem town, central Ethiopia. An institutional-based cross-sectional study was conducted from February to March 2016. Data were collected using pretested structured interviewer-administered questionnaires.

**Results:**

The mean age of the participants, 56.2% of whom were male was 25 (SD ± 5) years. The overall prevalence of work-related stress was 40.4% (95% CI 35.7, 45.3). Poor organizational support (adjusted odds ratio (AOR) 2.40, 95% CI 1.39, 4.77), inadequate work experience (AOR 3.77, 95% CI 1.68, 8.45), poor salary (AOR 7.04, 95% CI 3.39, 14.59), long working hours (AOR 3.40, 95% CI 2.00, 5.79), overtime work (AOR 2.24, 95% CI 1.10, 4.61), and poor physical environment (AOR 2.44, 95% CI 1.42, 4.19) were factors significantly associated with the stress. The prevalence of the stress was higher than what can be expected of many such employees. Poor organizational support, inadequate work experience, poor salary offers, long working hours, overtime work, and poor physical environment were significantly and independently associated with WRS. Establishing a functional collective agreement between employer and an employee trade union are needed to improve the problem.

**Electronic supplementary material:**

The online version of this article (10.1186/s13104-018-3727-5) contains supplementary material, which is available to authorized users.

## Introduction

Work-related stress occurs when there is a mismatch between the demands of the job and the resources and capabilities of the individual worker to meet those demands [1, 2]. Such stress is the second most reported work-related health problem next to musculoskeletal disorders [3–5]. The number of people suffering from stress-related conditions caused or made worse by work is increasing with an alarming rate and becoming an issue of public health concern in developing countries [6]. Globally, WRS accounts for 35% of all work related illnesses [7]. Studies conducted in Bristol City, England [8], Vietnam [9], Indian resident doctors [10], Iran [11], Tanzania [12], and Ethiopia [13] also show that one-in-three employees experience work-related stress.

Work-related stress is the major cause of occupational ill health which causes severe physical and psychological conditions resulting in poor productivity, human error, increased sick leaves, high staff turnover, and poor performance damaging the image of the organization both among workers and other stakeholders [2, 14].

According to research reports, some of the factors significantly associated with WRS include, over 50 h of work per week [15], long daily working hours [16], high work demands, time pressure, and too many administrative tasks [17].

Studies which primarily focused on the prevalence and associated factors in health care settings indicate that WRS is a major public health problem in developing countries. In Ethiopia, little is known about the magnitude and the determinants of the problem on the most at risk manufacturing industry workers. Therefore, this study aimed to assess work-related stress and its associated factors among Huajian shoe manufacturing employees in Dukem town, central Ethiopia. The information to be obtained is expected to increase the awareness of managers to prevent its occurrence, carryout early screening and detection of stress, and improve employee quality of life and organizational performance.

## Main text

### Methods

An institution based cross sectional study was conducted from February to March 2016, at Huajian Shoe Manufacturing Company, central Ethiopia. The company is located in Dukem town, 37 km southeast of Addis Ababa, the capital of Ethiopia. Owned by foreign investors, the company has around 4500 Ethiopian workers.

Employees currently engaged in the company and have worked for more than 6 months prior to the study were included in the study. Night shift workers during the study were excluded from the study. Sample size was determined using the single population proportion formula, assuming the proportion of work related stress to be 50%, level of certainty 95%, margin of error 5%, and non-response rate of 10%. The simple random sampling technique was used to select the 422 study participants.

A structured questionnaire was used to collect data on socio-demographic characteristics. Other previously validated tools employed for the purpose were the Work Place Stress Scale (WPSS) [18], the Job Content Questionnaire (JCQ) [19], and the National Institute for Occupational Safety and Health (NIOSH) [20]. These tools inquired about organizational factors (working conditions, job security, salary offer, experience in the current organization, working hours, organizational support, employee recognition, and overtime work), and job content factors (time pressure, job demand, job control, resources, opportunity to learn, interactions of people with machines, illness and physical environment).

To assess work-related stress, a 20-item standard questionnaire with a 5-point Likert scale was used. The scores ranged from 1 (Never) to 5 (Very often), and the reverse scores ranged from 5 (Never) to 1 (Very often) according to their perceived work place occurrence [13]. The results were summed for all WPSS questions, and a score below 60 was classified as having a work-related stress. Poor working conditions were considered when the summed scores of participants’ were less than 10. Poor organizational support was considered when the summed scores of participants’ were less than 7. High time pressure was defined as the summed scores of participants’ were more than 10. Poor physical environment was defined as the summed score of participants scores were below 9.

The questionnaires was initially prepared in English and translated to Affan Oromo (local language) and retranslated to English by language experts. Four health professionals and a supervisor participated in the data collection after two days intensive training. Data were checked for completeness, clarity, and consistency (Additional file 1).

The data entered using Epi Info version 7 and analyzed by SPSS version 20. Data were presented in the form of texts and tables. Bivariate logistic regression analysis was done and variables with p-values of up to 0.20 were included in the multivariable analysis to control possible effects of confounders. Variables which had significant associations with WRS were identified on the basis of OR with a 95% CI and p-values < 0.05.

### Results

A total of 406 participants were involved in the study with a response rate of 96%. The mean age of the participants was 25 (SD ± 5) years of whom 228 (56.2%) were male. Two hundred eleven (52%) were married; 214 (52.7%) had above secondary school education; 162 (39.9%) were Orthodox Christians, and 143 (35.2%) earned Ethiopian Birr (ETB) ≤ 1100/month (Table 1).

**Table 1:** Socio demographic characteristics of participants, Huajian shoe manufacturing, Dukem town, central Ethiopia, 2016 (n = 406)

Variables	Frequency	Percent
Sex		
Male	228	56.2
Female	178	43.8
Age		
18–21	61	15.0
22–26	208	51.2
27–30	104	25.6
31–34	31	7.6
≥ 35	2	0.5
Marital status		
Single	186	45.8
Married	211	52.0
Others^a^	9	2.2
Educational status		
Primary school	1	0.2
Secondary school	191	47.1
Above secondary	214	52.7
Religion		
Orthodox	162	39.9
Protestant	152	37.4
Muslim	71	17.5
Others^b^	21	5.2
Monthly income (ETB)^c^		
≤ 1100	143	35.2
1101–1300	91	22.4
1301–1450	102	25.1
> 1450	70	17.2
Type of employment		
Permanent	405	99.8
Temporary	1	0.2

^a^Divorce, widowed, separated

^b^Wakefeta, catholic

^c^Quartile classification

Of the participants, 234 (57.6%) got poor support from their organization; 279 (68.7%) worked in poor working conditions; 270 (66.5%) believed there was job insecurity; 219 (53.9%) got no recognition, and 211 (52%) were poorly paid for their work.

Regarding working hours, 214 (52.7%) of the participants worked ≤ 48 h/week, and 311 (76.6%) worked overtime for more than 20 h/month. Two hundred forty-nine (61.3%) and 261 (64.3%) of the participants experienced high time pressure and high attention job demands relating to their jobs, respectively. Of the total participants, 392 (96.6%) had poor learning opportunities; 104 (25%) had poor interaction with their machines, and 209 (51.5%) worked under a poor physical environment (Table 2).

**Table 2:** Organizational and Job related characteristics of participants, Huajian shoe manufacturing, Dukem town, central Ethiopia, 2016 (n = 406)

Variables	Frequency	Percent
Organizational support		
Good	172	42.4
Poor	234	57.6
Working conditions		
Good	127	31.3
Poor	279	68.7
Job security		
Good	136	33.5
Poor	270	66.5
Employees’ recognition		
Good	187	46.1
Poor	219	53.9
Salary offers		
Satisfied	195	48.0
Dissatisfied/poor	211	52.0
Work experience in the current organization in years^a^		
0.06–1.05	113	27.8
1.06–2.01	102	25.1
2.02–2.09	91	22.4
> 2.09	100	24.6
Working hours per week^b^		
≤ 48	214	52.7
> 48	192	47.3
Overtime working hours per month^b^		
≤ 20	95	23.4
> 20	311	76.6
Time pressure		
High	249	61.3
Low	157	38.7
Job demand attention		
High	261	64.3
Low	145	35.7
Illness		
Yes	355	87.4
No	51	12.5
Job control		
High	359	88.4
Low	47	11.6
Resource		
Enough	182	44.8
Scarcity	224	55.2
Learning opportunity		
Good	14	3.4
Poor	392	96.6
Interaction of people with machine		
Good	302	74.4
Poor	104	25.4
Physical environment		
Good	197	48.5
Poor	209	51.5

^a^Quartile classification

^b^Based on Ethiopian labour proclamation 377/2003

The overall prevalence of work-related stress was 40.4% (95% CI 35.7–45.3). The prevalence was higher, that is, 68.3%, 75%, and 78% among employees who worked under poor working physical environment, poor organizational support, and poor salary offers, respectively.

The multivariable logistic regression analysis revealed that poor organizational support, poor salary offers, inadequate work experience, over working hours, overtime work, and poor physical environments were significantly associated with work related stress. Accordingly, the likelihood of developing WRS for employees with payments of ETB ≤ 1100 ETB and between ETB 1101 and 1300 monthly were about six times (AOR 5.87, 95% CI 2.39, 14.42) and three times (AOR 3.02, 95% CI 1.19, 7.67) as those getting greater than ETB 1450, respectively.

Employees who worked under poor organizational support were two times (AOR 2.40, 95% CI 1.39, 4.77) as likely to develop WRS as those working under good organizational support. Employees who reported poor salary offers were seven times (AOR 7.04, 95% CI 3.39, 14.59) as likely to develop WRS as those with good salary offers. Employees who had 1.6–2.01 years of work experiences were about four times (AOR 3.77, 95% CI 1.68, 8.45) as likely to have WRS as those with work experiences of greater than 2.09 years.

Employees who worked for more than 48 normal working hours per week were three times (AOR 3.40, 95% CI 2.00, 5.79) as likely to have WRS as those working for 48 h or less per week. Employees who reported more than 20 overtime working hours per month were two times (AOR 2.24, 95% CI 1.10, 4.61) as likely to have work related stress as those working 20 h or less per month. Employees who worked under poor physical environments were two times (AOR 2.44, 95% CI 1.42, 4.19) as likely to have a work related stress as those working in good physical environments (Table 3).

**Table 3:** Bivariate and multivariable logistic regression analysis of factors associated with Work related stress (n = 406)

Variables	Work related stress		COR (95% CI)	AOR (95% CI)
	Yes	No		
Monthly income (ETB)				
≤ 1100	86	57	7.29 (3.60, 14.80)	5.87 (2.39, 14.42)*
1101–1300	39	52	3.63 (1.70, 7.70)	3.02 (1.19, 7.67)*
1301–1450	27	75	1.74 (0.81, 3.73)	1.13 (0.45, 2.89)
> 1450	12	58	1	1
Organizational support				
Good	41	131	1	1
Poor	123	111	3.54 (2.30, 5.50)	2.40 (1.39, 4.17)*
Salary offers				
Satisfied	36	159	1	1
Dissatisfied/poor	128	83	6.81 (4.32, 10.74)	7.04 (3.39, 14.59)*
Work experience in the current organization in years				
0.06–1.05	61	52	5.34 (2.85, 10.04)	1.99 (0.90, 4.39)
1.06–2.01	54	48	5.13 (2.69, 9.73)	3.77 (1.68, 8.45)*
2.02–2.09	31	60	2.35 (1.21, 4.59)	1.65 (0.72, 3.74)
> 2.09	18	82	1	1
Working hours per week				
≤ 48	55	227	1	1
> 48	109	83	3.80 (2.50, 5.77)	3.40 (2.00, 5.79)*
Overtime working hours per month				
≤ 20	17	74	1	1
> 20	147	164	4.11 (2.31, 7.27)	2.24 (1.10, 4.61)*
Job demand attention				
Low	129	107	1	1
High	126	135	2.63 (1.69, 4.09)	0.50 (0.24, 1.06)
Physical environment				
Good	52	145	1	1
Poor	112	97	3.22 (2.12, 4.89)	2.44 (1.42, 4.19)*

* p-value < 0.05; the Hosmer and Lemeshow test: 0.401

### Discussion

Work stress is recognized world-wide as a major challenge to employees’ health and the healthiness of their organizations. In this study, the overall prevalence of work-related stress was 40.4% (95% CI 35.7, 45.3). The result was comparable with those of studies conducted at Addis Ababa public hospitals (37.8%) [13] and West Sussex (43%) [21].

However, it was higher than those of studies done in European countries (35%) [7], Bristol City (20%) [8], Vietnam (20.7%) [9], India (32.8%) [10], Iran (21.3%) [11], and Tanzania (30.1%) [12]. The difference may be explained by the fact that developed countries with their better socio-economic status have organized safety precautions and facilitated access to health and safety trainings ahead of time. They have also improved the levels of enforcement regulations on health service delivery better than developing countries [5].

In this study, associations were observed between work-related stress and different variables. For example, poor organizational support had a significant association with WRS. The finding was consistent with those of studies conducted in Nigeria [22] and Sweden [23], where the likelihood of work-related stress was higher among employees with poor organizational support. The reason could be that employees with inadequate support from their employers often suffer from frustration, apathy, and poor performance. This in turn leads to unsafe work practices, staff turnover rise and even illness [24].

Poor salary offers had a significant association with-work related stress in this study. The finding was similar with that of a study conducted in Iran [11]. The reason for poor payment might be the availability of cheap labour and low market competitions in the country. Working for poor payments could increase stress due to the inability of employees to meet monthly obligations (supporting themselves and their families) [25].

Employees who had between 1.06 and 2.01 years of work experience in the current organization had a significant association with work related stress. This is due to the fact that the interaction of people with machines in the first stage and getting new incur results stress on their work.

Working for more than 48 working hours per week had a significant association with work related stress. The finding was consistent with those of studies conducted in Saudi Arabia [15], Germany and Austria [16], and the USA [26]. This is perhaps due to the fact that long working hours without enough breaks to attend to other life matters lead to exhaustion and affect health by impairing employee’s possibilities for sufficient recovery both mentally and physiologically [27, 28].

Working for more than 20 overtime working hours per month had a significant association with work related stress. The finding was supported by studies conducted in Tenibiaje Dele Joseph in Nigeria [4], Japanese workers [29], and in the United States [30] which stated that overtime work significantly increased the risk for work-related stress. This might be because overtime work prolongs high workload, interferes with leisure activities, causes too much employee physically and mentally fatigue to perform to the best of their ability, thereby increasing stress hormones [31].

Working under poor physical environments has a strong and independent role in increasing the likelihood of WRS. Consistent to what was reported from Ghana [32], Kerala [33], and Iran [34], this study revealed the likelihood of developing WRS was higher among employees who worked under poor physical environments. This is due to the fact that the elements of poor physical environments (unpleasant sound, extremely high or low temperature, poor air circulation, exposure to dangerous substances, smelling, and lighting) directly affect employee motivation and job performance adversely [35]. Such a persistent exposure to environmental stressors causes the immune system to be compromised; as a result, employees may have a more difficult time staying healthy and become stressed [36].

In conclusion, this study showed that a high proportion of employees had work-related stress. Poor organizational support, inadequate work experience, poor salary offers, over working hours, overtime work, and poor physical environment were significantly and positively associated with work-related stresses. Thus, employers need to attempt to update employee payments and implement reward evaluation systems, cut down the need for overtime or employ extra staff. Efforts should also be made to make the working environment conducive for workers and to minimize physical hazards that lead employees to work-related stress.

## Limitations

Since the data was collected in day time, we didn’t include night shift workers; this might have under estimated the proportion of the work-related stress.

## Additional file

**Additional file 1.** WRS-Questionnaires. This is the English version of the questionnaires.

## Abbreviations

AOR: adjusted odds ratio
ETB: Ethiopian Birr
JCQ: Job Content Questionnaire
NIOSH: National Institute for Occupational Safety and Health
OR: odds ratio
WPSS: Work Place Stress Scale
WRS: work-related stress
