# Supplementary material for: Work related stress and associated factors among Huajian shoe manufacturing employees in Dukem town, central Ethiopia
Source: BMC Res Notes. 2018 Aug 24;11:610. doi: 10.1186/s13104-018-3727-5 (PMC6109341; doi:10.1186/s13104-018-3727-5)
Supplement: Supplementary file 1 — Additional file 1. WRS-Questionnaires. This is the English version of the questionnaires. [file 13104_2018_3727_MOESM1_ESM.docx]

**Part I: Socio- Demographic Characteristics (please circle your appropriate response)**

| No. | Questions | Response | | | | | | | | | Remark |
| --- | --- | --- | --- | --- | --- | --- | --- | --- | --- | --- | --- |
|  | Sex | 1. Male 2. Female | | | | | | | | |  |
|  | Age | _________year | | | | | | | | |  |
|  | Marital Status | 1. Single 2. Married 3. Widowed 4. Divorced 5. Separated | | | | | | | | |  |
|  | Current educational status | 1. Illiterate 2. Primary school (1-8) 3. Secondary school (9-12) 4. Above secondary (TVET, degree...) | | | | | | | | |  |
|  | Religion | 1. Orthodox  2. Muslim  3. Protestant  4. Others | | | | | | | | |  |
|  | Monthly income | _____________ Birr. | | | | | | | | |  |
|  | Type of employment | 1. Permanent  2. Temporary | | | | | | | | |  |
| **Part II : Organizational risk factors**  For each statement, please circle the number to indicate your degree of agreement/ sensation | | | | | | | | | | | |
| No. | Statements | Strongly disagreed | | Disagree | | | Neither | | agree | | Strongly agree |
| Organizational support | |  | |  | | |  | |  | |  |
|  | Do you feel close to the people at work? | 1 | | 2 | | | 3 | | 4 | | 5 |
|  | Do you get along with your supervisors and manager? | 1 | | 2 | | | 3 | | 4 | | 5 |
| Working conditions | |  | |  | | |  | |  | |  |
|  | Do you believe management is concerned about your health and safety? | 1 | | 2 | | | 3 | | 4 | | 5 |
|  | Do you feel good about working at this company? | 1 | | 2 | | | 3 | | 4 | | 5 |
|  | Do you believe work is good for your physical health? | 1 | | 2 | | | 3 | | 4 | | 5 |
|  | Organizational job security |  | |  | | |  | |  | |  |
|  | Do you feel secure about your job? | 1 | | 2 | | | 3 | | 4 | | 5 |
|  | Do you feel good at your job? | 1 | | 2 | | | 3 | | 4 | | 5 |
|  | Employees recognition |  | |  | | |  | |  | |  |
|  | Do you received recognition for a job well done? | 1 | | 2 | | | 3 | | 4 | | 5 |
|  | Do you feel all your talents and skills are used at work? | 1 | | 2 | | | 3 | | 4 | | 5 |
|  | Organizational salary offers |  | |  | | |  | |  | |  |
|  | Do you believe wages are good? | 1 | | 2 | | | 3 | | 4 | | 5 |
|  | Do you feel that the reward or incentive systems are good? | 1 | | 2 | | | 3 | | 4 | | 5 |
|  | Questions | | Response | | | | | | | | Remark |
|  | How long have you been working in your current organization? | | ____ years and ____ months | | | | | | | |  |
|  | How many hours do you normally work per week in your job? | | _________Hrs./week | | | | | | | |  |
|  | How many hours overtime do you work in your job in an average month? (Please mark “0” if no overtime) | | _________Hrs./month | | | | | | | |  |
| **Part III: Questions on the job related factors**  Thinking about your job and describe how often you feel? Then circle the number to indicate you feeling | | | | | | | | | | | |
|  | **Statements** | | Never | | Rarely | | Sometimes | | Fairly Often | | Very often |
|  | Time pressure | |  | |  | |  | |  | |  |
|  | How often does your job require you to work *very fast*? | | 5 | | 4 | | 3 | | 2 | | 1 |
|  | How often does your job require you to work *very hard*? | | 5 | | 4 | | 3 | | 2 | | 1 |
|  | How often does your job leave you with *little* time to get things done? | | 1 | | 2 | | 3 | | 4 | | 5 |
|  | High attention demand | |  | |  | |  | |  | |  |
|  | How often is there a *great deal* to be done? | | 5 | | 4 | | 3 | | 2 | | 1 |
|  | How often is there a marked increase in the work load? | | 5 | | 4 | | 3 | | 2 | | 1 |
|  | How often is there a marked increase in the amount of concentration required on your job? | | 5 | | 4 | | 3 | | 2 | | 1 |
|  | **Illness** | |  | |  | |  | |  | |  |
|  | You were in ill health which affected your work? | | 5 | | 4 | | 3 | | 2 | | 1 |
|  | **Job control** | |  | |  | |  | |  | |  |
|  | Can you exercise control in your position? | | 1 | | 2 | | 3 | | 4 | | 5 |
|  | Can you control the quality of what you produce? | | 1 | | 2 | | 3 | | 4 | | 5 |
|  | **Resource** | |  | |  | |  | |  | |  |
|  | Do you have enough resource in your team? | | 1 | | 2 | | 3 | | 4 | | 5 |
|  | **Learning opportunity** | |  | |  | |  | |  | |  |
|  | Do you think the work environment is encouraging and incites learning? | | 1 | | 2 | | 3 | | 4 | | 5 |
|  | **Interaction people with machine** | |  | |  | |  | |  | |  |
|  | Do you think that you can interact well with the machine ? | | 1 | | 2 | | 3 | | 4 | | 5 |
| No. | **Physical conditions** | | Response | | | | | | | | Remark |
|  | The level of **NOISE** in the area in which you work is usually high? | | - 1. Yes   2. No | | | | | | | |  |
|  | The level of **lighting** in the area in which you work is usually poor? | | - - 1. Yes     2. No | | | | | | | |  |
|  | The **temperature** of your work area is usually uncomfortable? | | - - - 1. Yes       2. No | | | | | | | |  |
|  | The level of **air circulation** in your work area is poor? | | yes  No | | | | | | | |  |
|  | The **air** in your work area is smelling unpleasant? | | 1. Yes  2. No | | | | | | | |  |
|  | In your job, are you exposed to **dangerous substances**? | | 1. Yes  2. No | | | | | | | |  |
| **Part IV: WORK-RELATED STRESS QUESTIONNAIRE**  The following questions are important that your responses reflect your work in the last six months | | | | | | | | | | | |
| No. | Statements | | Never | | | Rarely | | Sometimes | | Fairly Often | Very often |
|  | Are you clear what is expected from you at work? | | 1 | | | 2 | | 3 | | 4 | 5 |
|  | Can you decide when to take a break? | | 1 | | | 2 | | 3 | | 4 | 5 |
|  | Different groups at work demand things from you that are hard to combine? | | 5 | | | 4 | | 3 | | 2 | 1 |
|  | Do you know how to go about getting your job done? | | 1 | | | 2 | | 3 | | 4 | 5 |
|  | Are you subjected to personal harassment in the form of unkind words or behavior? | | 5 | | | 4 | | 3 | | 2 | 1 |
|  | Do you have unachievable deadlines? | | 5 | | | 4 | | 3 | | 2 | 1 |
|  | If work gets difficult, do your colleagues will help you? | | 1 | | | 2 | | 3 | | 4 | 5 |
|  | Are you clear what your duties and responsibilities are? | | 1 | | | 2 | | 3 | | 4 | 5 |
|  | You have to neglect some tasks because you have too much to do? | | 5 | | | 4 | | 3 | | 2 | 1 |
|  | Can you talk to your line manager about something that has upset or annoyed you about work? | | 1 | | | 2 | | 3 | | 4 | 5 |
|  | Did you received respect at work you deserve from your colleagues? | | 1 | | | 2 | | 3 | | 4 | 5 |
|  | Have you got supportive feedback on the work what you do? | | 1 | | | 2 | | 3 | | 4 | 5 |
|  | Do you think your working time can be flexible? | | 1 | | | 2 | | 3 | | 4 | 5 |
|  | Do your colleagues are willing to listen to your work-related problems? | | 1 | | | 2 | | 3 | | 4 | 5 |
|  | Do you have some say over the way you work? | | 5 | | | 4 | | 3 | | 2 | 1 |
|  | Pressure at work causes you to come to work when you are not well enough to work? | | 5 | | | 4 | | 3 | | 2 | 1 |
|  | Pressure at work causes you to do your job less well? | | 5 | | | 4 | | 3 | | 2 | 1 |
|  | Have you taken time off due to pressure at work? | | 5 | | | 4 | | 3 | | 2 | 1 |
|  | Have you consider leaving this organization due to pressure at work? | | 5 | | | 4 | | 3 | | 2 | 1 |
|  | Pressure at work has affected your health whilst working in this organization? | | 5 | | | 4 | | 3 | | 2 | 1 |
